# Predicting neuropsychiatric symptoms of persons with dementia in a day care center using a facial expression recognition system

**DOI:** 10.18632/aging.203869

**Published:** 2022-02-03

**Authors:** Liang-Yu Chen, Tsung-Hsien Tsai, Andy Ho, Chun-Hsien Li, Li-Ju Ke, Li-Ning Peng, Ming-Hsien Lin, Fei-Yuan Hsiao, Liang-Kung Chen

**Affiliations:** 1 Aging and Health Research Center, Taipei, Taiwan; 2 Institute of Public Health, National Yang-Ming Chiao-Tung University, Taipei, Taiwan; 3 Center for Geriatrics and Gerontology, Taipei, Taiwan; 4 uAge Day Care Center, Taipei Veterans General Hospital, Taipei, Taiwan; 5 Value Lab, Acer Incorporated, New Taipei City, Taiwan; 6 Graduate Institute of Clinical Pharmacy, National Taiwan University, Taipei, Taiwan; 7 School of Pharmacy, National Taiwan University, Taipei, Taiwan; 8 Department of Pharmacy, National Taiwan University Hospital, Taipei, Taiwan; 9 Taipei Municipal Gan-Dau Hospital, Taipei, Taiwan

**Keywords:** artificial intelligence, behavioral and psychological symptoms of dementia, dementia, facial expression recognition system, machine learning

## Abstract

Background: Behavioral and psychological symptoms of dementia (BPSD) affect 90% of persons with dementia (PwD), resulting in various adverse outcomes and aggravating care burdens among their caretakers. This study aimed to explore the potential of artificial intelligence-based facial expression recognition systems (FERS) in predicting BPSDs among PwD.

Methods: A hybrid of human labeling and a preconstructed deep learning model was used to differentiate basic facial expressions of individuals to predict the results of Neuropsychiatric Inventory (NPI) assessments by stepwise linear regression (LR), random forest (RF) with importance ranking, and ensemble method (EM) of equal importance, while the accuracy was determined by mean absolute error (MAE) and root-mean-square error (RMSE) methods.

Results: Twenty-three PwD from an adult day care center were enrolled with ≥ 11,500 FERS data series and 38 comparative NPI scores. The overall accuracy was 86% on facial expression recognition. Negative facial expressions and variance in emotional switches were important features of BPSDs. A strong positive correlation was identified in each model (EM: *r* = 0.834, LR: *r* = 0.821, RF: *r* = 0.798 by the patientwise method; EM: *r* = 0.891, LR: *r* = 0.870, RF: *r* = 0.886 by the MinimPy method), and EM exhibited the lowest MAE and RMSE.

Conclusions: FERS successfully predicted the BPSD of PwD by negative emotions and the variance in emotional switches. This finding enables early detection and management of BPSDs, thus improving the quality of dementia care.

## INTRODUCTION

Behavioral and psychological symptoms of dementia (BPSDs), or neuropsychiatric symptoms, affect 90% of persons with dementia (PwD) over the course of their illness and are associated with greater morbidity, mortality, and distress between caretakers and their family members [1, 2]. BPSDs may present in 50–60% of PwD and 40–50% of persons with mild cognitive impairment [3]. Agitation, psychosis, depression, and apathy were the dominant clusters of BPSDs and were key factors in initiating the diagnosis of dementia [4]. Moreover, unmitigated BPSDs can accelerate cognitive declines, functional limitations, mistreatment, the use of physical/chemical restraints, and premature institutionalization [4, 5]. Currently, the main BPSD management includes early identification and individualized, interdisciplinary, and nonpharmacological interventions instead of pharmacological approaches [2].

The potential of artificial intelligence (AI)-based facial expression analysis using a facial expression recognition system (FERS) to identify emotions, pain, and nonverbal information among persons with psychiatric disorders has been documented [6–9]. FERS successfully predicted 8 basic mood phenotypes using more than 1,000,000 facial images collected from the internet, i.e., disgust, fear, sadness, anger, happiness, surprise, neutral, and contempt [9–11]. The accuracy of FERS based on a convolutional neural network (CNN) to recognize these 8 emotional expressions was approximately 87.7-94.2%, which was noninferior to that of the support vector machine (SVM) method (77.1-92.8%) [9–11]. Although deficient facial expressions were common presentations of persons with neurodegenerative disorder, the enhanced facial responses to pain in PwD provided opportunities for FERS to identify somatic discomforts [8, 10, 12]. The advanced development of AI technology and deep learning programs enables FERS to identify facial expressions and their changes over time from video streams, creating opportunities to develop the automatic detection of BPSDs to improve the quality of dementia care [7–10, 13]. Evidence suggests that BPSDs are often related to suboptimal management of physical pain, but pain is not the only aggravating factor that precipitates or aggravates BPSDs in PwD [14]. Currently, the evaluation of BPSDs in PwD is dependent on informant-based interviews, which is not only time-consuming but also influenced by inter- and intrarater reliability and recall bias from caretakers. Hence, this study aimed to develop a system combining customized FERS and AI analytics algorithms to predict BPSDs and severity among PwD to improve the process of BPSD management and the quality of dementia care.

## RESULTS

Overall, data from 23 participants (median age: 83.6 years, IQR: 78.4–88.2 years; 34.7% males) were obtained for analysis. Approximately half (56.5%) of the participants were diagnosed with Alzheimer’s disease, with a median Mini-Mental State Examination (MMSE) score of 18 (IQR: 11.5–20), Clinical Dementia Rating Scale (CDR) score of 1 (IQR: 1–2), and Neuropsychiatric Inventory (NPI) score of 2 (IQR: 0–12) (Table 1). Among all participants, more than 11,500 facial expression data series were collected with 38 corresponding Neuropsychiatric Inventory (NPI) scores from 23 participants, and data were divided into two groups (Stage 1 and Stage 2) based on their time of recruitment for analysis. The model accuracy was 86% for facial expression identification by the customized FERS for all participants. Comparisons of demographic characteristics, multimorbidity, and the prevalence of neuropsychiatric symptoms between groups (Stage 1 and Stage 2) are shown in Table 1. Compared to the Stage 1 samples, the Stage 2 samples had a higher prevalence of agitation/aggression (100% vs. 71.4%, *p* = 0.025) but a lower prevalence of sleep disorders (62.5% vs. 100%, *p* = 0.059).

**Table 1:** Demographic characteristics, cognitive performance, prevalence of neuropsychiatric symptoms and multimorbidity among the participants.

	**Overall (n=23)**	**Stage 1 (n=7)**	**Stage 2 (n=16)**	***p* value**
**Age (years)**	83.6 (78.4-88.2)	85.7 (74.1-88.2)	83.2 (78.5-88.2)	0.871
**Male gender, n (%)**	8 (34.7%)	3 (42.9%)	5 (31.2%)	0.525
**BMI (kg/m^2^)**	24.6 (21.7-28.3)	23.7 (20.6-27.5)	25.3 (21.6-29.1)	0.541
**Education, n (%)**				0.157
≤ 6 years	10 (43.4%)	2 (28.6%)	8 (50.0%)	
> 6 years	13 (56.5%)	5 (71.4%)	8 (50.0%)	
**Dementia subtype, n (%)**				0.506
Alzheimer disease	13 (56.5%)	5 (71.4%)	8 (50.0%)	
Vascular dementia	10 (43.4%)	2 (28.6%)	8 (50.0%)	
**Dementia severity by CDR**	1 (1-2)	2 (0.75-2.5)	1 (1-2)	0.622
**Cognition by MMSE**	18 (11.5-20)	18 (2.75-20.25)	18 (12-20)	0.791
**Neuropsychiatric symptoms by NPI**	2 (0-12)	0 (0-25)	3.5 (1-12)	0.624
Delusion, n (%)	21 (91.3%)	6 (85.7%)	15 (93.8%)	0.529
Hallucination, n (%)	19 (82.6%)	6 (85.7%)	13 (81.3%)	0.795
Agitation/Aggression, n (%)	21 (91.3%)	5 (71.4%)	16 (100%)	0.025*
Dysphoria, n (%)	11 (47.8%)	5 (71.4%)	6 (37.5%)	0.134
Anxiety, n (%)	17 (73.9%)	5 (71.4%)	12 (75.0%)	0.858
Euphoria, n (%)	23 (100%)	7 (100%)	16 (100%)	1.000
Apathy, n (%)	22 (95.6%)	7 (100%)	15 (93.8%)	0.499
Disinhibition, n (%)	21 (91.3%)	6 (85.7%)	15 (93.8%)	0.529
Irritability/Liability, n (%)	19 (82.6%)	5 (71.4%)	14 (87.5%)	0.349
Aberrant motor activities, n (%)	18 (78.2%)	5 (71.4%)	13 (81.3%)	0.599
Sleep disorder, n (%)	17 (73.9%)	7 (100%)	10 (62.5%)	0.059
Intake disorder, n (%)	20 (86.9%)	7 (100%)	13 (81.3%)	0.219
**Depression by GDS-5**	1 (0-2)	1 (0.5-2)	0 (0-2)	0.444
**Multimorbidity by CCI**	1 (1.5-2.25)	1.5 (1-3.25)	1.5 (1-2)	0.802

*p < 0.05, **p < 0.01, ***p < 0.001.

Abbreviation: BMI, body mass index; CCI, Charlson's comorbidity index; CDR, Clinical dementia rating scale; GDS-5, Geriatric depression scale-5 items; MMSE, Mini-mental status examination; NPI, Neuropsychiatric inventory.

Among all 23 participants, a strong positive correlation was found on the frequency of negative mood (*r* = 0.98), sadness (*r* = 0.93), anger (*r* = 0.77), and frequency of mood switches (*r* = 0.83) in the last 7 days, while a strong negative association was noted among differences between positive and negative moods (i.e. differences (Happiness-Sadness) (*r* = -0.84) and the mean amplitude of moods (*r* = -0.87) by stepwise linear regression (LR) (Table 2). The frequency of sadness, anger, neutrality, standard deviation of emotional presentation within 7 days, and the range of mood switches were candidate features for NPI score prediction by stepwise LR in both stages. The results showed that frequency of sadness, negative emotions, differences between positive and negative moods, mean amplitude, and standard deviation of facial expression phenotypes within 7 days were potential features ranked 1^st^–6^th^ by random forest (RF) with importance in both stages (Table 2). In both prediction models, negative facial expressions and variance among emotions were identified as important features to predict the NPI of PwD.

**Table 2:** Association between facial expression phenotype and scores of neuropsychiatric symptoms among persons with dementia.

**Facial expression phenotypes**	**Stage 1 (n=7)**					**Stage 2 (n=16)**				**Overall (n=23)**
	**Stepwise linear regression**			**Random forest**		**Stepwise linear regression**			**Random forest**	***r***
	**β ± SE**	***p* value**		**Ranking of importance**		**β ± SE**	***p* value**		**Ranking of importance**	
**Frequency of specific mood**										
**Sadness**	9.52 **±** 0.34	<0.001***		1		10.85 **±** 2.86	<0.001***		3	0.93
**Anger**	15.80 **±** 0.52	<0.001***		7		7.70 **±** 5.70	0.185		8	0.77
**Negative (Sadness + Anger)**	N.A.	N.A.		2		N.A.	N.A.		2	0.98
**Neutrality**	-3.72 **±** 0.43	0.003**		8		0.66 **±** 0.41	0.116		6	-0.66
**Happiness**	N.A.	N.A.		10		5.35 **±** 2.70	0.055		9	-0.40
**Differences (Happiness - Sadness)**	N.A.	N.A.		5		N.A.	N.A.		4	-0.84
**Mean amplitude of moods**	8.82 **±** 1.05	0.003**		4		N.A.	N.A.		1	-0.87
**Standard deviation of moods**	-38.15 **±** 5.17	0.005**		6		-36.12 **±** 20.92	0.092		5	0.65
**Frequency of mood switches**	N.A.	N.A.		3		-3.53 **±** 2.00	0.086		7	0.83
**Range of mood switches**	5.43 **±** 1.02	0.013*		9		16.56 **±** 7.95	0.044*		10	0.68

*p < 0.05, **p < 0.01, ***p < 0.001.

Abbreviation: N.A, not-available; NPI, neuropsychiatric inventory; SE, standard error.

The accuracy of different models for NPI score prediction in PwD was assessed by the mean absolute error (MAE) and root-mean-square error (RMSE) methods (Table 3), and the ensemble method (EM) of equal importance showed the best performance with the lowest MAE (1.641 and 4.868) and RMSE (2.348 and 7.547) in both stages. Although the performance of the RF in NPI score prediction remained stable in both Stage 1 and Stage 2, the performance of the LR and EM in Stage 2 was not as good as that in Stage 1 (Table 3).

**Table 3:** Predictive accuracy of the severity of neuropsychiatric symptoms between different models.

**Predictive accuracy**	**Stage 1**			**Stage 2**		
	**Ensemble method**	**Linear regression**	**Random forest**	**Ensemble method**	**Linear regression**	**Random forest**
MAE	1.641	3.738	5.237	4.868	6.591	5.182
RMSE	2.348	6.962	10.227	7.547	10.598	9.020

Abbreviations: MAE, mean absolute error; RMSE, root-mean-square error.

The patientwise sampling method and MinimPy sampling method were performed on data from all 23 participants in the final model. A strong positive correlation was identified between predictive scores and true NPI scores in each prediction model (EM: *r* = 0.834, LR: *r* = 0.821, RF: *r* = 0.798 by the patientwise method; EM: *r* = 0.891, LR: *r* = 0.870, RF: *r* = 0.886 by the MinimPy method). The performance of the EM was still better than that of the other 2 methods, exhibiting the lowest MAE (EM: 4.333, LR: 4.967, RF: 4.917 by the patientwise method; EM: 3.749, LR: 4.356, RF: 4.000 by the MinimPy method) and RMSE (EM: 8.491, LR: 8.786, RF: 9.391 by the patientwise method; EM: 5.497, LR: 6.209, RF: 6.087 by the MinimPy method) (Table 4). Comparisons between the true NPI scores and the predictive scores from the EM, LR, and RF models in PwD are presented in detail (Table 5).

**Table 4:** Predictive accuracy of the severity of neuropsychiatric symptoms by different sampling methods on all participants.

**Predictive accuracy**	**Patient-wised**			**MinimPy**		
	**Ensemble method**	**Linear regression**	**Random forest**	**Ensemble method**	**Linear regression**	**Random forest**
**MAE**	4.333	4.967	4.917	3.749	4.356	4.000
**RMSE**	8.491	8.786	9.391	5.497	6.209	6.087
***r***	0.834	0.821	0.798	0.891	0.870	0.886
***p* value**	< 0.001***	< 0.001***	< 0.001***	0.001**	0.003**	0.002**

*p < 0.05, **p < 0.01, ***p < 0.001.

Abbreviations: MAE, mean absolute error; RMSE, root-mean-square error.

**Table 5:** Comparing the true neuropsychiatric scores and the predicting scores between different models.

**Stages**	**Cases**	**True NPI score**	**Predictive score by ensemble method**	**Predictive score by linear regression**	**Predictive score by random forest**
**Stage 1**	A	0	1.93	0	3.87
	B	50	44.9	66.5	23.4
	C	25	28.1	32.9	23.2
	D	0	0.42	0	0.83
	E	12	11.9	13.6	10.2
	F	0	0.45	0	0.9
	G	0	0.42	0	0.86
**Stage 2**	H	0	0	0	1
	I	36	28	30	26
	J	0	4	6	2
	K	0	1	3	0
	L	9	6	5	7
	M	1	6	9	4
	N	8	25	28	23
	O	0	0	0	0
	P	5	11	12	11
	Q	0	4	6	2
	R	40	47	61	34
	S	0	0	0	0
	T	0	2	3	2
	U	2	5	9	1
	V	30	30	26	34
	W	14	9	13	5

Abbreviation: NPI, Neuropsychiatric inventory.

## DISCUSSION

To the best of our knowledge, this is the first study combining customized FERS and deep learning algorithms to predict BPSDs in PwD based on facial expressions, and the EM provided a superior approach to predict NPI scores with better accuracy. The results of this study clearly demonstrated the potential of applying FERS with AI-based algorithms to detect BPSDs early and the temporal changes in NPI scores to improve the quality of dementia care. Effective interpersonal communication relies on multiple elements, and facial expressions were the most important (55%), followed by voice tone (38%) and words (5%) [10]. Although PwD progressively lost their verbal communication ability, the ability to express emotions through facial activities was maintained even in moderate to severe dementia [15]. Existing evidence indicates that enhanced facial expressions related to pain in PwD are detectable by FERS and may be an effective approach to alleviate somatic or psychological discomfort among people with limited communication ability [16–18]. Socioemotional disinhibition may intensify the inner response toward noxious stimuli or stressful situations, which is associated with the loss of cortical volume in the orbitofrontal regions among persons with neurodegenerative disorders [12, 19, 20]. Therefore, the facial expressions related to NPI scores in PwD may originate from certain specific neurodegenerative processes and neuropathology.

This study showed that video-based FERS provided an effective nonverbal tool to identify neuropsychiatric symptoms of PwD and may be employed in different clinical settings to identify medical conditions, psychiatric disorders, or neurological diseases, including dementia [8, 13]. As a noninvasive and less costly instrument, FERS with AI-based algorithms also reduced the concerns of inter- and intrarater variation in the interview-based manually scored NPI [10, 13]. The accuracy of the customized, video-based FERS was 86% in the Karolinska Directed Emotional Faces (KDEF) dataset; the results were comparable to those developed by histograms of oriented gradients (HOG) of facial feature extraction and other convolutional neural network (CNN) models (Table 6) [10, 21–26].

**Table 6:** Performance comparison between different studies with the Karolinska Directed Emotional Faces dataset.

**Method**	**#Expression**	**Accuracy (%)**
Our study	7	86.0
ExpNet: CNN [21]	7	71.0
VGG-Face Deep Convolutional Network model [22]	7	72.6
HOG+SRC [23]	7	82.2
SCAE+CNN [24]	7	92.5
LeNet-5: CNN [25]	7	90.6
DeepExp3D: CNN [26]	7	92.4

Abbreviations: CNN, convolutional neural network; HOG, histogram of oriented gradient method; SCAE, Stacked Convolutional Auto-Encoder; SRC, Sparse representation classifier; SVM, Support Vector Machine.

Negative emotions, including sadness and anger, as well as emotional variance, were important characteristics to predict BPSDs in this study, also suggesting unmet care needs in PwD. Irritability/liability, agitation/aggression, and anxiety in BPSD pose an enormous caretakers’ burden and are positively associated with negative emotions in their facial expressions [4, 27]. The facial expressions in persons with Alzheimer’s disease or schizophrenia were impaired to some extent, so the greater emotional excursion in these patients suggested worsened socioemotional disinhibition and more advanced neurodegeneration [4, 8, 12, 19, 20, 27, 28]. Facial expression disturbance is an important feature for persons with depression, anxiety, and other psychiatric disorders that may effectively be captured by the video-recorded eye tracking approach [10, 13]. A previous study showed the benefits of emotion-oriented care planning with nonpharmacological interventions in reducing stress reactions in PwD [29].

Compared to traditional informant-based interviews, FERS with AI-based algorithms clearly demonstrated its advantages in promoting the quality of dementia care. First, the AI-based approach eliminated the potential bias related to the inter- and intrarater variation from the informant-based interviews. NPI scores were sometimes overestimated due to the psychological stress of the primary caregivers, highlighting the strength of AI-based assessments. Second, the current use of psychotropic agents was recorded as the most severe BPSD in the NPI, but families were sometimes reluctant to withhold these medications due to worries about symptom recurrence or rebound. In this case, the facial expressions captured by FERS with AI algorithms may rarely represent the severity of BPSDs. Third, the prediction model of FERS was derived from the image data series within the last 7 days, providing more real-time information compared to the traditional NPI scores using information from the past 30 days. Compared to informant-based interviews, FERS substantially reduced the recall bias in assessing BPSDs of PwD and facilitated proactive modification of care plans.

Despite all efforts spent in this study, there were still several limitations. First, the NPI scores selected as outcome indicators were ordinal variables in nature, but the relationships between specific facial expressions and NPI scores may be nonlinear. The prediction model might be influenced by the uneven distribution of neuropsychiatric symptom severity between participants in a small sample-sized study. Second, collinearity among specific facial expressions may influence the development of prediction models, such as sadness, anger, and their sum. The nonlinear outcome indicators and collinearity among potential predictors partially explained the differences in the performance by LR between stages 1 and 2. However, the performance of RF remained stable in both stages, and the EM model was even better in its accuracy. Hence, the effects of the abovementioned factors were less influential in the model development. Third, delusion, hallucination, sleep disturbance, and intake disorder in PwD were inner-conflicting conditions that were not easily detected by facial expressions, which may explain the discrepancy between the true NPI scores and predictive values by FERS.

Combining customized FERS and AI-based algorithms, the neuropsychiatric symptoms of older people with dementia were successfully predicted with high accuracy. This noninvasive, cost-saving approach provided an effective and efficient method to assess BPSDs in PwD, so caretakers were able to modify the care plans for care recipients in a timely manner. The prediction models developed by FERS identified negative emotions more than other domains in the NPI, but the model remained stable and robust by subset data analysis. Further studies conducted with larger sample sizes and longer observational periods or in places outside day care centers are needed to externally validate the prediction model and to develop the process of care planning based on the FERS results.

## MATERIALS AND METHODS

Persons with dementia looked after in an adult day care center in Taiwan were invited to participate in the study from October 2019 to May 2020. Participants were screened by the following criteria: age ≥ 65 years, having a confirmed diagnosis of dementia, and having an expected life expectancy of ≥ one year. Care recipients were excluded if they had the following conditions: a serious and uncontrolled medical illness, being unable to respond properly during assessments, stopping service utilization in 6 months, or declining video recording for data analysis. For all eligible participants, data on baseline demographic characteristics, dementia diagnosis and severity, neuropsychiatric symptoms, and performance of serial functional assessments were collected for analysis.

Cognitive performance was assessed by the MMSE-Traditional Chinese version with a maximal score of 30, and lower MMSE scores indicated poorer cognitive function [30]. In addition, the CDR was used to rate the dementia severity into categories such as very mild (0.5), mild (1), moderate (2), or severe (3) was adopted to evaluate the dementia severity in this study [31]. Depressive symptoms were assessed by the Geriatric Depression Scale-5 item version (GDS-5), and a higher GDS-5 score suggested more depressive symptoms [32]. Moreover, the multimorbidity of all participants was assessed by the Charlson Comorbidity Index (CCI) [33].

The frequency and severity of BPSDs were evaluated by the NPI-Traditional Chinese edition. The NPI is a comprehensive and validated instrument to assess BPSDs, which includes 12 domains of delusion, hallucination, agitation/aggression, dysphoria/depression, anxiety, euphoria/elation, apathy/indifference, disinhibition, irritability/liability, aberrant motor behavior, nighttime behavior, and appetite/eating disorder in the last month. A higher NPI score indicates more severe BPSDs and greater care stresses [34].

**Figure 1:**
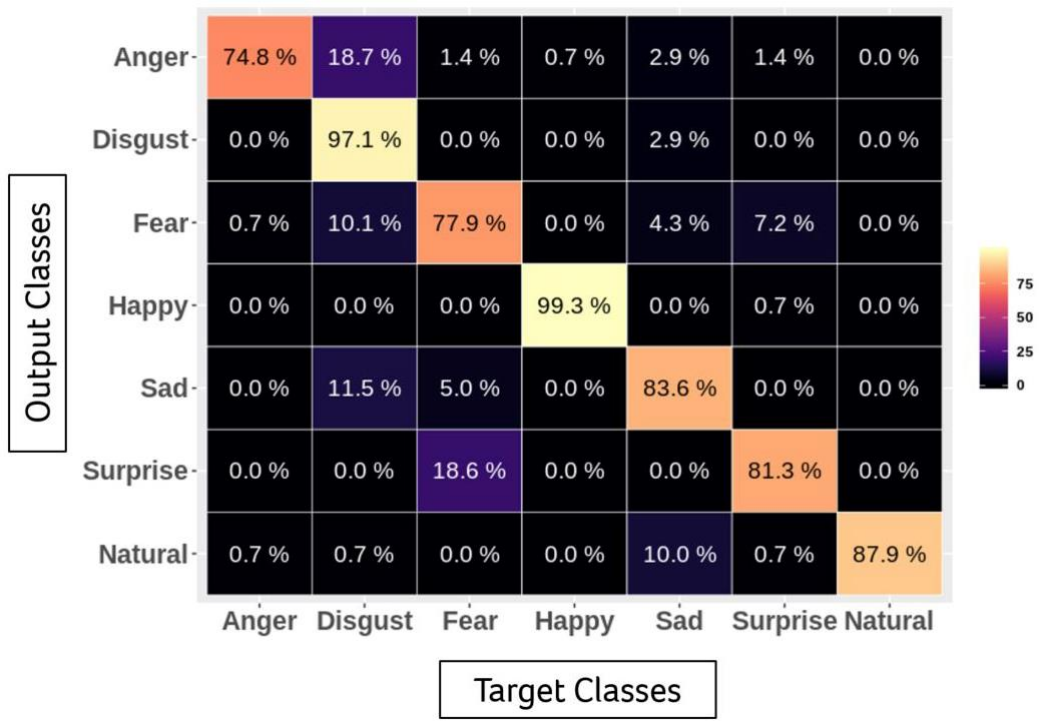
Accuracy of emotional classification by facial expression recognition system.

To train a multitask model to classify emotion and other facial features, such as gender and age, simultaneously, we self-collected and labelled a large-scale dataset including more than 100,000 face images. Before training, we aligned all images by similarity transformation according to the centermost face and its five facial landmarks detected by RetinaFace. Each aligned image is 112 × 112. To meet the requirements of both accuracy and inference speed on embedded hardware, we applied MobileFaceNet as the backbone of the classification model and customized the last layer to three classifiers for different usages (emotion and other facial features) [35]. To evaluate the accuracy of our model, we used the Karolinska Directed Emotional Faces database (KDEF), a public dataset containing 70 subjects with 7 basic emotion annotations (happy, sad, fearful, angry, surprised and disgusted) pictured from 5 different angles, as the benchmark. Only the subsets of frontal images (980 in total) were applied for the evaluation. The performance of the confusion matrix is presented in the following table (Figure 1).

**Figure 2:**
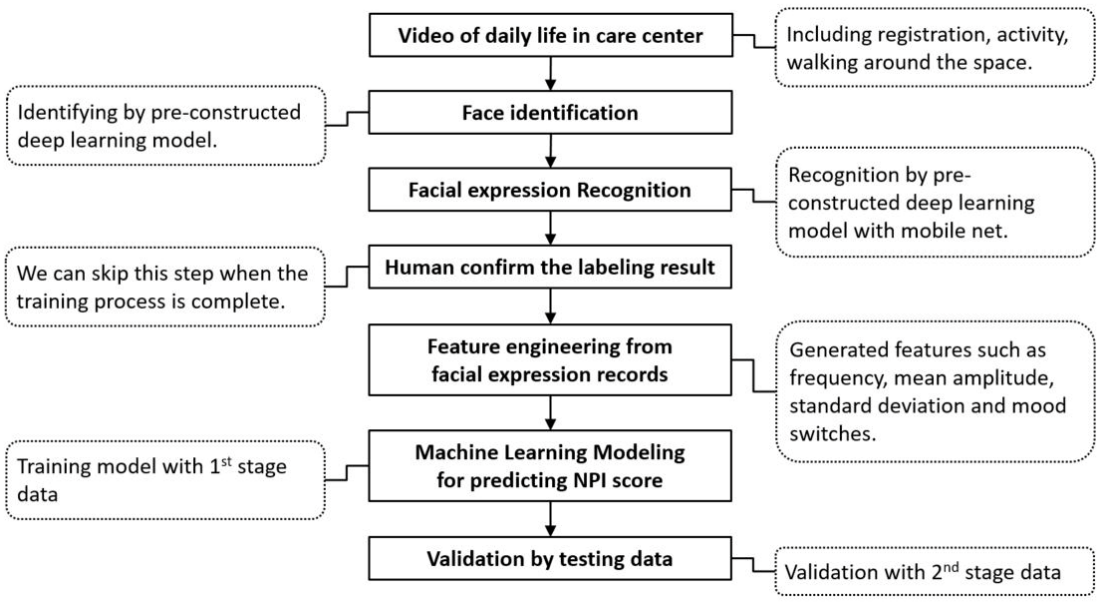
Flow chart of the two-stage training process for predicting the NPI score with facial expression data.

Figure 2 shows the training process for NPI score prediction by deep learning. All participants were divided into two groups (stages 1 and 2) based on their time of recruitment. In Stage 1, half of the data from 7 randomly selected participants were used for model training to identify candidate features for the prediction models, and the other half of the data were utilized for validation. In Stage 2, the same data processing for model training and testing was applied based on the data series from the other 16 participants for internal validation.

After collecting the video of daily activities in the day care center, the customized FERS identified 4 basic phenotypes of mood presentations, including negatives of anger and sadness, neutrality, and positives of happiness. Facial expression phenotypes in the past 7 days prior to the NPI assessments were registered and assigned ordinal numbers (anger = 1, sadness = 2, neutrality = 3, happiness =4) to establish possible predictive models through feature engineering and data mining. The frequency of specific moods, mean amplitude, standard deviation, and differences between positive and negative emotions in the last 7 days were features generated from the facial expression data for machine learning algorithm modeling, as well as the frequency and range of mood switches. An individual’s facial expression records in N days were considered as E1, E2, E3… and EN. To predict the value of the NPI score, Ei, i=1, 2, 3…, and N can be considered ordinal variables. We used the facial expressions to generate the following numerical variables (Table 7).

**Table 7:** Methods for features registration among participants.

**Features**	**Description**	**Formula**
(1)Frequency of negative emotion (sadness + anger)	Count the number of sadness and anger in N days	$\sum_{i=1}^{N}T_{i}$, where $T_{i}=\{\begin{array}{cc} 1, & \text{if }E_{i}\;\text{is sad or angry}\\ 0, & \text{otherwise} \end{array}$
(2)Frequency of sadness	Count the number of sadness in N days	$\sum_{i=1}^{N}T_{i}$, where $T_{i}=\{\begin{array}{cc} 1, & \text{if }E_{i}\;\text{is sad }\\ 0, & \text{otherwise} \end{array}$
(3)Frequency of anger	Count the number of anger in N days	$\sum_{i=1}^{N}T_{i}$, where $T_{i}=\{\begin{array}{cc} 1, & \text{if }E_{i}\;\text{is angry}\\ 0, & \text{otherwise} \end{array}$
(4)Frequency of neutrality	Count the number of neutrality in N days	$\sum_{i=1}^{N}T_{i}$, where $T_{i}=\{\begin{array}{cc} 1, & \text{if }E_{i}\;\text{is neutral}\\ 0, & \text{otherwise} \end{array}$
(5)Frequency of happiness	Count the number of happiness in N days	$\sum_{i=1}^{N}T_{i}$, where $T_{i}=\{\begin{array}{cc} 1, & \text{if }E_{i}\;\text{is smile}\\ 0, & \text{otherwise} \end{array}$
(6)Frequency of difference (happiness - sadness)	Counts difference between happiness and sadness in N days	(5)-(2)
(7)Mean amplitude of moods	Average of emotion in N days	$\frac{\sum_{i=1}^{N}E_{i}}{N}$
(8)Frequency of mood switches	Total quantity of emotion change in N days	$\overset{N}{\underset{i=2}{\sum }}\left|E_{i}-E_{i-1}\right|$
(9)Range of mood switches	Range of emotion change in N days	$\underset{i}{\max}\left|E_{i}-E_{i-1}\right|-$ $\underset{i}{\min}\left|E_{i}-E_{i-1}\right|$
(10)Standard deviation of moods	Standard deviation of emotion in N days	$\sqrt{\frac{\sum_{i=1}^{N}{\left(E_{i}-\bar{E}\right)}^{2}}{N-1}}$, where $\bar{E}=\frac{\sum_{i=1}^{N}E_{i}}{N}$

This study was approved by the Institutional Review Board of Taipei Veterans General Hospital (TPEVGH IRB No. 2019-08-006C), and it conformed to the provisions of the World Medical Association’s Declaration of Helsinki revised in 2000. Written informed consent from participants and their families was obtained prior to study enrollment after full explanation.

### Statistical analysis

Continuous variables are expressed as medians with interquartile ranges (IORs), and comparisons of continuous variables between groups used nonparametric Mann–Whitney U tests. Categorical variables are presented as percentages, and comparisons between groups were performed using the chi-square test on baseline characteristics. Linear regression (LR) with the stepwise selection method was performed among facial expression phenotypes with a high association with NPI scores, while potential candidates were presented as beta coefficients with standard errors if they were retained in the prediction model. Random forest (RF) with the importance ranking method was also employed to identify possible predictors among facial expression phenotypes for the NPI scores, and the prediction model was established using variables ranked 1^st^–6^th^ in both stages. The ensemble method (EM) was created by weighting the two prediction models of equal importance (EM = [LR + RF]/2). The accuracy for predicting NPI scores between the different models was compared by MAE and RMSE measures. Lower MAE and RMSE values indicated better accuracy in predictions.

Eventually, we performed advancing analysis on data from all participants by different sampling methods. Randomized patientwise sampling by patient and NPI level was performed, and we split data as 6-fold to evaluate the accuracy with cross validation. MinimPy sampling was also employed to split data into training or testing sets stratified by sex, age and NPI level [36]. All data analyses were performed with the R project for statistical computing software version 3.6.3 for Windows, and variables were considered statistically significant if *p* < 0.05.
